# FIH-1, a Novel Interactor of Mindbomb, Functions as an Essential Anti-Angiogenic Factor during Zebrafish Vascular Development

**DOI:** 10.1371/journal.pone.0109517

**Published:** 2014-10-27

**Authors:** Ju-Hoon So, Jun-Dae Kim, Kyeong-Won Yoo, Hyun-Taek Kim, Seung-Hyun Jung, Jung-Hwa Choi, Mi-Sun Lee, Suk-Won Jin, Cheol-Hee Kim

**Affiliations:** 1 Department of Biology, Chungnam National University, Daejeon, Korea; 2 Department of Developmental Biology, University of Pittsburgh, Pittsburgh, Pennsylvania, United States of America; 3 Yale Cardiovascular Research Center, Section of Cardiovascular Medicine, Department of Internal Medicine, Yale University School of Medicine, New Haven, Connecticut, United States of America; 4 School of Biological Sciences, Gwangju Institute of Science and Technology, Gwangju, Korea; Medical College of Wisconsin, United States of America

## Abstract

**Objective:**

It has been shown that Mindbomb (Mib), an E3 Ubiquitin ligase, is an essential modulator of Notch signaling during development. However, its effects on vascular development remain largely unknown.

**Approaches and Results:**

We identified a number of novel proteins that physically interact with Mib, including the Factor Inhibiting Hypoxia Inducible Factor 1 (FIH-1, also known as HIF1AN) from a yeast two hybrid screen, as previously reported. In cultured cells, FIH-1 colocalizes with Mib1, corroborating their potential interaction. In zebrafish embryos, FIH-1 appears to modulate VEGF-A signaling activity; depletion of *fih-1* induces ectopic expression of *vascular endothelial growth factor–a* (*vegfa*) and leads to exuberant ectopic sprouts from intersegmental vessels (ISVs). Conversely, over-expression of *fih-1* substantially attenuates the formation of ISVs, which can be rescued by concurrent over-expression of *vegfa*, indicating that FIH-1/HIF1AN may fine tune VEGF-A signaling.

**Conclusions:**

Taken together, our data suggest that FIH-1 interacts with Mib E3 Ubiquitin ligase and modulates vascular development by attenuating VEGF-A signaling activity.

## Introduction

During development, actively growing areas within embryos are exposed to hypoxic environments, and the vascular network undergoes a rapid expansion to accommodate an increasing demand of oxygen and nutrient supplies. To facilitate formation of blood vessels, cells experiencing hypoxia express Hypoxia Inducible Factor 1α (HIF1α), a transcription factor which in turn induces the expression of Vascular Endothelial Growth Factor-A (VEGF-A) [1]–[3]. While VEGF-A serves as the main factor that promotes proliferation, migration, and survival of endothelial cells (ECs), additional factors are shown to provide an essential role during vascular development [4], [5]. For instance, Bone Morphogenetic Proteins provide context-dependent pro- and anti-angiogenic effects [6], [7] EphrinB2/EphB4 signaling regulates the separation of arterial and venous ECs [8], and Angiopoietins regulate maturation of blood vessels [9]. In addition, the Notch pathway, which mediates lateral inhibition during development [10], appears to be an essential factor to ensure proper vascular development. Lack of Notch signaling causes failure of arterial specification [11], [12], ectopic tip cell formation which causes excessive angiogenic sprouting [13], as well as negatively modulating PFKFB3 expression to regulate metabolism of ECs [14].

Mind bomb (Mib1) is an E3 Ubiquitin ligase whose function is essential for Notch signaling [15]. Mib1 appears to be evolutionarily conserved [16], [17]and functionally related to *Drosophila* Neuralized, which promotes endocytosis of Delta [15]. From a large scale screen, mutations that affect the *mind bomb* (*mib*) locus have been identified in zebrafish which cause pronounced morphological defects in the anterior neural tube [15]. In addition, ECs within developing intersegmental vessels (ISVs) of *mib* ^−/−^ embryos generate exuberant secondary angiogenic sprouts [12], [18], suggesting that Mib1 may function as an anti-angiogenic factor during development. Previous reports suggest that the vascular defects of *mib* ^−/−^ embryos appear to be dependent on Notch signaling; first, Mib1 has been implicated in the processing of Delta, a key endogenous ligand for Notch [15]. Second, inhibition of Notch signaling causes excessive angiogenic sprouts within the ISVs, reminiscent of *mib* ^−/−^ embryos [13]. However, considering the promiscuity of E3 Ubiquitin ligases [19], it is likely that additional targets and/or interactors of Mib1 may contribute to the vascular phenotype in *mib* ^−/−^ embryos. Moreover, the vascular phenotype of *mib* ^−/−^ embryos are more severe than those with compromised Notch signaling [12], [18], suggesting that there may be additional factors that mediate Mib function during vascular development.

In this report, we identified novel interactors of Mib using a yeast two hybrid screen. Among potential interactors was Factor Inhibiting Hypoxia Inducible Factor 1 (FIH-1, also known as HIF1AN), which is an asparaginyl hydroxylase [20]. Previously, FIH-1 has been shown to regulate HIF1α by forming a ternary complex with an E3 Ubiquitin ligase, Von Hippel-Lindau tumor suppressor (VHL) [20]. Moreover, Tseng and colleagues have recently shown that FIH-1 forms a complex with Mib1 and Mib2 [21]. Inhibition of FIH-1 in zebrafish embryos substantially increases ectopic angiogenic sprouts from the ISVs, while over-expression of FIH-1 causes severe disruption in ISVs, suggesting that FIH-1 functions as an anti-angiogenic factor during development. Manipulation of VEGF-A can alleviate the vascular defects caused by FIH-1 over-expression. Considering that FIH-1 targets HIF1α, which induces the expression of VEGF-A [22], [23], our data suggest that FIH-1 may function as a mediator linking the anti-angiogenic function of Mib to VEGF-A, and serve as an essential modifier of signaling during vascular development.

## Experimental Procedures

### Zebrafish Maintenance

The animal study conducted here was performed under the protocol approved by Yale University IACUC committee (protocol # 2013–11402), and Chungnam University animal research ethics committee. Wild type, *Tg(kdrl:EGFP)^ s843^* [24], *Tg(hsp:vegfaa165)^ck4^;Tg(kdrl:EGFP)^s843^* and *mind bomb^ta52b^* mutant (*mib^ta52b^*) [15] zebrafish were raised and kept under standard laboratory conditions at 28.5°C. To better visualize internal structures, embryos were incubated with 0.2 mM 1-phenyl-2-thiourea (Sigma) to inhibit pigmentation and fixed at specific developmental stages [25].

### Yeast Two Hybrid Screening

A PCR fragment encoding the ankyrin repeats domain of Mib was cloned into the vector pGBT9 (Clontech) downstream from the GAL4 binding domain, pGBT9/Ank-MIB. After transformation of yeast host strain AH109 cells with pGBT9/Ank-MIB plasmid, the yeast cells were sequentially mated with Y187 yeast cells which were pre-transformed Human Matchmaker cDNA Libraries (Clontech). Positive colonies were selected on Trp-, Leu-, His-, and Ade-depleted plates, followed by a quantitative assay of β-galactosidase activity. Plasmid DNAs were purified from the candidate yeast colonies, and used as templates for PCR and sequencing.

### Preparation of DNA and mRNA for microinjection and anti-sense RNA probes

To isolate zebrafish *fih-1*, RT-PCR was performed with total RNA isolated from several stages of zebrafish embryos by using specific primers. Used primer sequences were 5′-AGAATTCAATGGCGGAGACCGACGGA-3′ as forward primer and 5′-ACTCGAGTTCAGTACAGGCTATTAACTCAG-3′ as reverse primer for isolating of full length of *fih-1*. The amplified fragment of the full-length zebrafish *fih-1* cDNA was subcloned into the pGEM-T easy vector and it was digested with EcoRI and XhoI and was transferred into full-length *fih-1* pCS2+ expression vector. To make capped mRNA, plasmids were linearized by NotI and transcribed with SP6 RNA polymerase using the mMESSAGE mMACHINE SP6 in vitro transcription kit (Ambion) according to the manufacturer's instructions. Full-length of *vegfaa_165_* (NM_131408.3) [26] PCR fragment was digested with EcoRI in pGEM T-easy vector (Promega), and transferred into expression vector pCS2+. Clones with sense orientation were confirmed by sequencing. Generation of capped mRNA was performed as described above. The *vegfaa_165_*, *hif1α*, and *vhl* templates for ribo-probes were generated by PCR using the following primers; *vegfaa_165_*: (forward) 5′ GAGAGCCAGCGACTCACCGCAACAC 3′, (reverse) 5′ GTTCGCTCGATCATCATCTTGGC 3′, *hif-1α*: (forward) 5′ TACTGAGTGGTTTCACCCAGG 3′, (reverse) 5′ CAGCTTCCTCTTACGCTGGAT 3′, *vhl* : (forward) 5′ GGATCCTCCTGTCTTTGACGATGCCC 3′, (reverse) 5′ GTTAATGTTGATGTTTTCGTCTG 3′. To synthesize digoxigenin (Roche)-labeled anti-sense RNA probes, plasmids containing *fih-1*, *kdrl* (NM_131472.1), *fli1a* (NM_131348.2), *hif1α* (NM_200233.1), and *vhl* (NM_001080684.1) were linearized by using restriction enzymes, respectively, prior to transcription with either T3, SP6 or T7 RNA polymerase (Fermentas). To preferentially detect *vegfaa165* message, anti-sense RNA probe was designed from the exon7 of the *vegfaa* gene.

### Microinjection of mRNA and morpholino oligonucleotides

*fih-1* (100pg) or *vegfaa_165_* mRNA (30pg or 120pg) were injected into 1–4 cell stage embryos. To confirm the injected volume, each injection solution was visualized by adding phenol red with final concentration 0.5%. Also, 200pg of *fih-1*ΔC or 80pg of *fih-1*ΔN was injected into *Tg(kdrl:EGFP)* 1–2 cell stage embryos. The sequence of the anti-sense mopholino (Gene Tools) used to target the exon 2 splice donor site of the *fih-1*gene was 5′-CATTAATCACACTGACATACCTTCC −3′. The sequence of the control MO was 5′-CCTCTTACCTCAGTTACAATTTATA −3′. Approximately 9 ng of *fih-1* or control MOs was injected into 1–2 cell stage embryos. Rescue experiments were performed by injecting 200 pg of *fih-1* full-length mRNA into *fih-1* MO-injected embryos.

### Cell cultures and transfection

COS7 cells (from ATCC) were maintained in DMEM medium containing 10% heat inactivated fetal bovine serum and anti-biotics. The cDNA coding for zebrafish *fih-1* was subcloned into pCS2+ EGFP via NcoI. *fih-1* pCS2+ EGFP was co-transfected with mouse HA-Mib1 pcDNA and/or HA-Hif1α pcDNA into the cultured cells. Cells were transfected with appropriate amounts of plasmid DNA using Lipofectamine Plus (Invitrogen).

### Treatment of VEGF receptor tyrosine kinase inhibitor with zebrafish embryo

5 µg/ml of VEGF receptor tyrosine kinase inhibitor (VEGFRTKI, Calbiochem) was treated in *fih-1* MO-injected *Tg(kdrl:EGFP)* embryos from 55 hpf to 80 hpf. Control *Tg(kdrl:EGFP)* embryos were treated in DMSO from 55 hpf to 80 hpf.

### Whole-mount in situ hybridization and Immunohistochemistry

Whole mount *in situ* hybridization was performed as previously reported [27]. For immunohistochemistry, COS7 cells were transfected with various plasmids. At 24 hours post-transfection, the cells were washed in PBS and fixed in 4% paraformaldehyde for 30 min at 4 C. The fixed cells were incubated in blocking solution (3% skim milk and 0.1% Triton X-100 in PBS) overnight at 4 C, and then stained with appropriate primary antibody in 3% skim milk in PBS for 1 h at room temperature. Subsequently, the cells were incubated with an anti-mouse antibody conjugated with Rhodamine for 30 min at room temperature. For DNA staining, cells were stained with Hoechst 33342 for 5 min. After rinsing with 0.1% Triton X-100 in PBS, the cells were mounted on glass slides and analyzed with a Leica DM5000B fluorescent microscope.

### Microangiography

Microangiography was performed as previously reported [28]. FITC-Dextran with a molecular weight of 2000 kDa (Sigma) was used for microangiography. FITC-Dextran was solubilized in Danieau solution and this solution injected into the sinus venosa/cardinal vein of the anaesthetized 48 h embryo.

### Generation of *Tg(hsp70l:vegfaa165)* ^ck4^ transgenic zebrafish

A 1.5 kb heat-shock 70 promoter cloned into *vegfaa_165_* pCS2+. The plasmid was linearized by SacII and microinjected into *Tg(kdrl:EGFP)* embryos of 1–2 cell stage. Embryos were maintained in egg water and screened for germ-line transmitted founders by whole-mount *in situ* hybridization with *vegfaa_165_* ribo-probe. Transgene positive f1 lines were identified by whole-mount *in situ* hybridization with *vegfaa_165_* and used to establish the *Tg(hsp70l:vegfaa165)^ck4^* line.

### Heat-shock treatment of *Tg(hsp70l:vegfaa165)* ^ck4^

Wild-type and heterozygous double *Tg(hsp70l:vegfaa165)^ck4^* embryos at 50 hpf were collected and heat-shocked by placing in a 37°C incubator for 1 hour and then observed under the fluorescence microscope at 80 hpf (Leica, DM5000B).

### Quantitative Real-time PCR

Control and *fih-1* MOs were injected at 1–2 cell stage, and resulting embryos were collected at 48 hpf to extract total RNA using RNeasy Mini Kit (Qiagen). 1 µg total RNA was used to generate cDNA using High-Capacity cDNA Reverse Transcription Kit (Applied Biosystems). Quantitative real-time reaction was performed using Fast SYBR Green Master Mix Real-Time PCR Master Mix (Applied Biosystems). Following primers were used: *vegfaa* forward primer, 5′-TGCTGGTAGACATCATCCA-3′; *vegfaa* reverse primer, 5′-TTATGCTGCGATACGCGTT-3′; *gapdh* forward primer, 5′-AGCACTGTTCATGCCATCA-3′; *gapdh* reverse primer, 5′-GTCAGATCCACAACAGAGA-3′.

### BrdU (5-bromo-2-deoxyuridine) incorperation and whole-mount immunostaining

Dechorionated embryos were incubated at 28.5°C in 10 mM BrdU (Sigma)/15% Dimethylsulfoxide (DMSO) for 20 minutes. After incubation, embryos were fixed for 2 hours at room temperature in 4% paraformaldehyde. Embryos were washed four times by PBS with 0.1% tween 20 (PBST) for 5 minutes and incubated in 10 µg/ml proteinase K (Roche) for 10 minutes. Embryos were re-fixed in 4% paraformaldehyde for 20 minutes and washed with H_2_O, then incubated in 2N HCL for 1 hour. Then, embryos were blocked in 5% horse serum for 1 hour. Next, embryos were incubated with anti-BrdU antibody (Sigma, 1∶1000) in for 2 hours at room temperature. Subsequently, embryos were incubated for overnight at 4°C with Rhodamine-conjugated anti-mouse secondary antibody (Molecular Probe, 1∶500), and washed with PBST. Photographs were taken on a Zeiss LSM5.

## Results

### FIH-1 physically associates with Mindbomb

**Figure 1:**
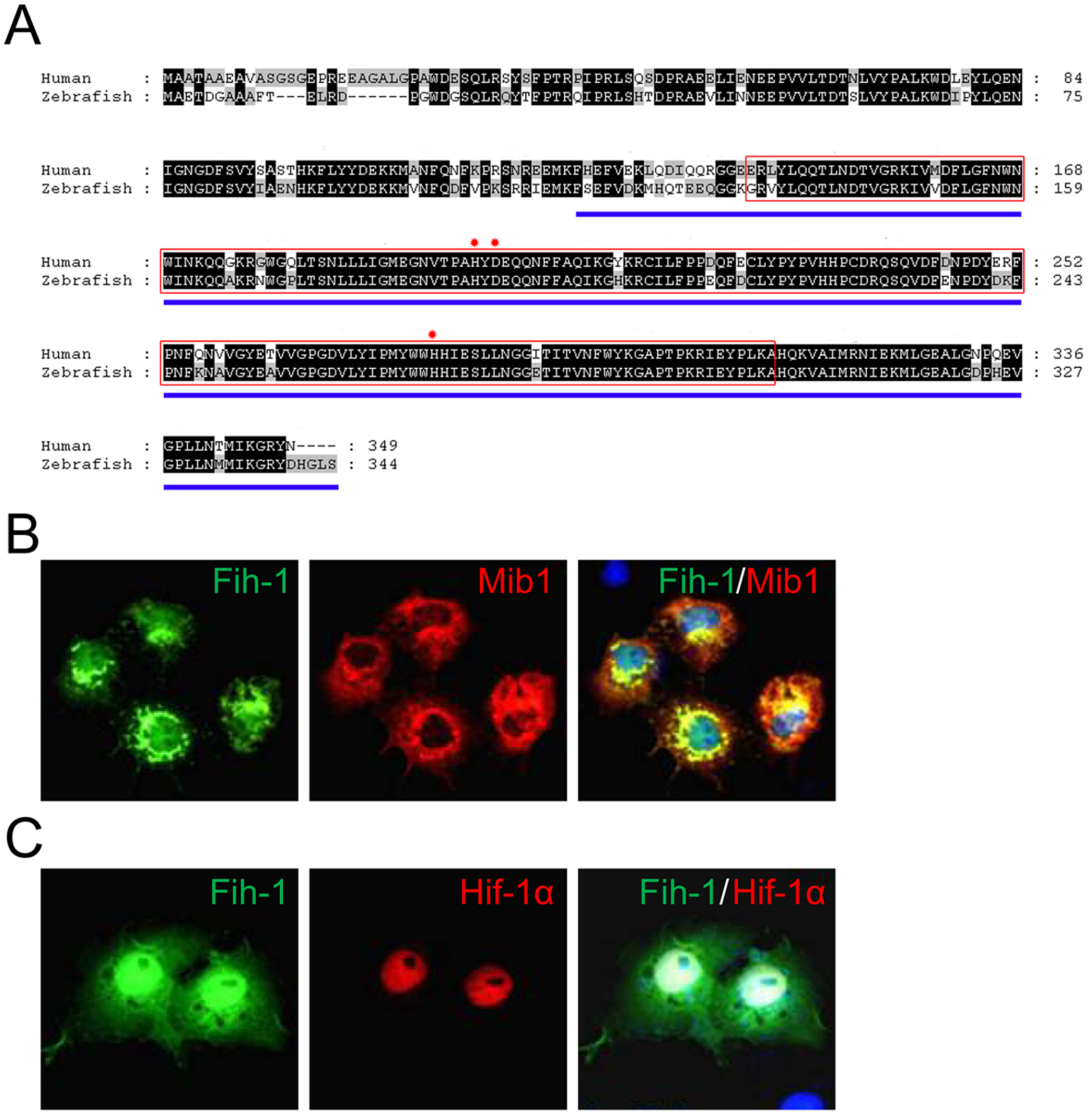
Association of Factor Inhibiting HIF-1α (FIH-1) with Mindbomb. (**A**) Amino acid sequence comparison between human and zebrafish FIH-1/Fih-1. JmjC domain is marked as a red box. Region of FIH-1 which mediates its interaction with HIF-1α is marked as blue underline. Red asterisks indicate metal coordination sites. (**B**) Zebrafish Fih-1 co-localizes with Mib1 in transiently transduced Cos7 cells. (**C**) Zebrafish Fih-1 co-localizes with Hif1α in transiently transduced Cos7 cells. Expression vectors encoding HA-Mib1 and Fih-1-GFP (For B) or HA-Hif-1α and Fih-1-GFP (For C) were used.

To identify interactors of Mib1, we performed yeast two-hybrid screens using the Ankyrin repeats of Mib1 (Fig. 1A and Fig. S1). From a screening of 1.44×10^6^ colonies of cDNA library, we have isolated a number of candidates that physically interact with Mib1 (Fig. 1B), including SNX5 [29], RanBP9, and MAP1A. In addition, FIH-1, also known as HIF1AN, a protein that negatively modulates HIF-1α activity [20], [23] was also identified as a potential interactor of Mib1.

FIH-1 appears to be a highly conserved protein; human and zebrafish FIH-1 are 91 percent similar and 79 percent identical. Specifically, amino acid 133 to 301 which roughly corresponds to the Jumanji (JmjC) domain, has over 96 percent homology, illustrating the importance of this domain for FIH-1 function (Fig. 1C, red box). Previously, FIH-1 has shown to associate with Mib and its paralog Mib2, and undergoes Mib/Mib2-mediated Ubiquitination [21]. Consistent with this report, we find that FIH-1 and Mib1 appear to colocalize in the perinuclear region in cultured cells which were transfected with FIH-1-GFP and HA-Mib1 (Fig. 1B). In addition, FIH-1 appears to colocalize with HIF-1α while FIH-1 is also known to interact with HIF-1α (Fig. 1C) [20].

**Figure 2:**
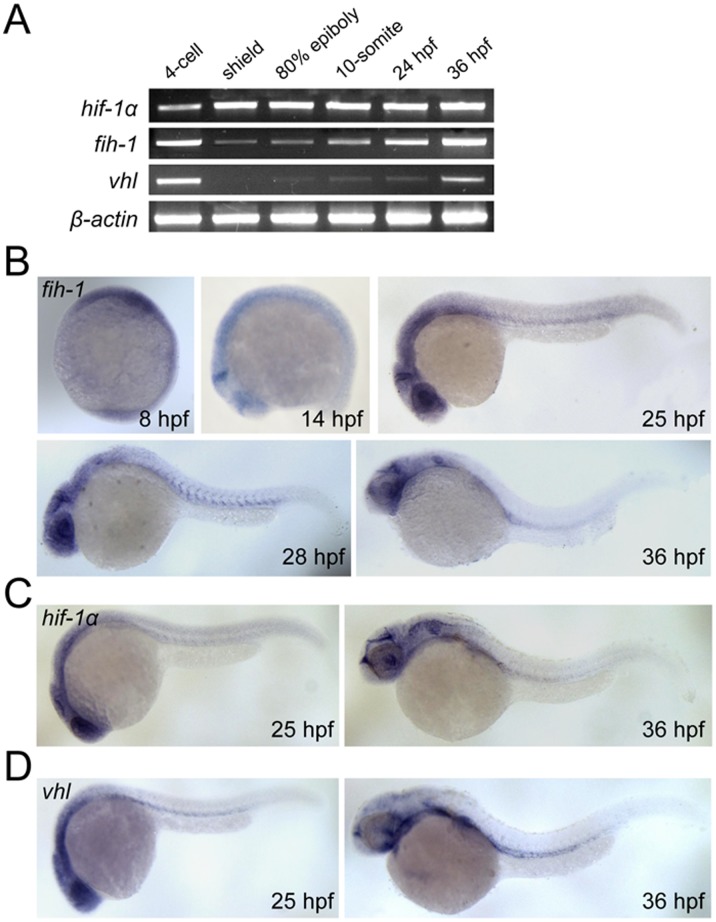
Expression pattern of *fih-1*, *vhl*, and *hif-1α*. (**A**) RT-PCR analysis showing expression of *fih-1*, *hif-1a*, *vhl*, and *β-actin* during development. (**B**) Whole-mount *in situ* hybridization of *fih-1* during development. As early as 8 hpf, *fih-1* expression can be detected. At 14 hpf, *fih-1* is strongly expressed at the midbrain-hindbrain boundary and eye. At 25 hpf, expression of *fih-1* is expanded to the optic vesicle and ventral mesoderm. The expression of *fih-1* in midbrain-hindbrain boundary, eye, optic vesicle and ventral mesoderm is maintained at later stages. (**C**) Expression of *hif1α*, a known target of Fih-1, at 25 hpf and 36 hpf. (**D**) Expression of *vhl*, which is known to interact and synergize with Fih-1, at the same developmental stage. Both *hif1α* and *vhl* express within the similar anatomical region as *fih-1*.

During development, expression of *fih-1/hif1an* can be detected as early as the 4-cell stage, suggesting that *fih-1* may be maternally deposited (Fig. 2A). Its expression subsequently decreases until 24 hours post-fertilization (hpf), which is reminiscent of *vhl* expression, another well-characterized inhibitor of HIF-1α [30], [31]. While *fih-1* is widely expressed in developing embryos, its expression is gradually restricted to the ventral mesoderm, midbrain hindbrain boundary, and eye at later stages (Fig. 2B). The expression pattern of *fih-1* at later stages (25 and 36 hpf) is comparable to those of *vhl* and *hif1a* (Fig. 2C and 2D), further illustrating the functional relationship between FIH-1, VHL, and HIF-1 during development (Fig. 2A).

### Inhibition of FIH-1 leads to increased angiogenic activity in zebrafish embryos

**Figure 3:**
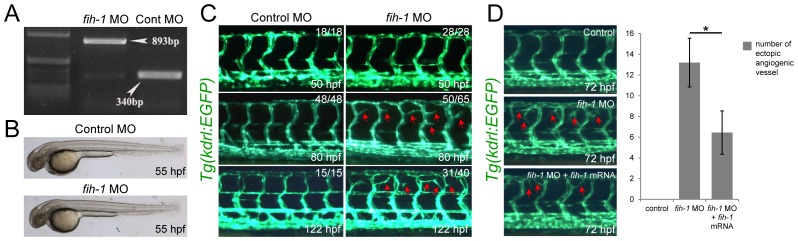
Fih-1 negatively modulates angiogenesis during development. (**A**) Validation of the morpholino (MO) targeting *fih-1*. Injection of *fih-1* MO interferes with mRNA splicing, leading to the retention of an intron and premature translation termination. (**B**) Gross morphology of fih-1 MO-injected embryos in comparison with control MO-injected embryos. No obvious gross morphological defects were observed at 55 hpf. (**C**) At 50 hpf, injection of *fih-1* MO did not cause any obvious increase in angiogenic sprouts, however, exuberant angiogenic sprouts emerge from the intersegmental vessels (ISVs) in *fih-1* MO-injected embryos at 80 and 122 hpf. (**D**) Concomitant injection of *fih-1* mRNA can rescue exuberant angiogenesis caused by *fih-1* MO-injected embryos. On the right column, control (top, n = 12), *fih-1* MO (middle, n = 20), or *fih-1* MO and *fih-1* mRNA (bottom, n = 20) injected embryos. Arrows point to ectopic angiogenic sprouts. Quantification of ectopic angiogenic sprouts per embryo is shown on the right. Asterisk notes statistical significance in the number of ISVs between *fih-1* MO and *fih-1* MO and *fih-1* mRNA injected embryos. Error bars are standard deviation.

Considering its proposed role as a negative modulator of HIF-1α signaling [20], FIH-1 may regulate vascular development. To examine this possibility, we first injected a morpholino (MO) targeting *fih-1* into developing zebrafish embryos (Fig. 3A). At 12 hpf, inhibition of *fih-1* did not cause any discernible changes in the expression of early EC makers, such as *fli-1a* and *scl* (Fig. S2). At 55 hpf, *fih-1* MO-injected embryos were morphologically comparable to control MO-injected embryos (Fig. 3B). To better elucidate the function of FIH-1 during vascular development, we examined the morphology of the developing vascular structure in *Tg(kdrl:EGFP)* transgenic zebrafish [24]. At 50 hpf, when the initial sprouting of ISVs from the dorsal aorta has been completed, we did not observe any obvious difference between control and *fih-1* MO-injected embryos. However, at this time point, proliferation rate of endothelial cells in *fih-1* MO-injected embryos had been substantially increased, suggesting that Vegf-A signaling may be up-regulated (Fig. S3). Subsequently, approximately 80 and 122 hpf, ectopic secondary sprouts become emanate from the ISVs in *fih-1* MO-injected embryos (Fig. 3C, red arrows). The exuberant secondary sprouts appear to be caused by a reduced level of FIH-1 activity since co-injection of *fih-1* mRNA is able to rescue this phenotype (Fig. 3D).

### FIH-1 negatively regulates VEGF-A165 in a HIF-1α-dependent manner

**Figure 4:**
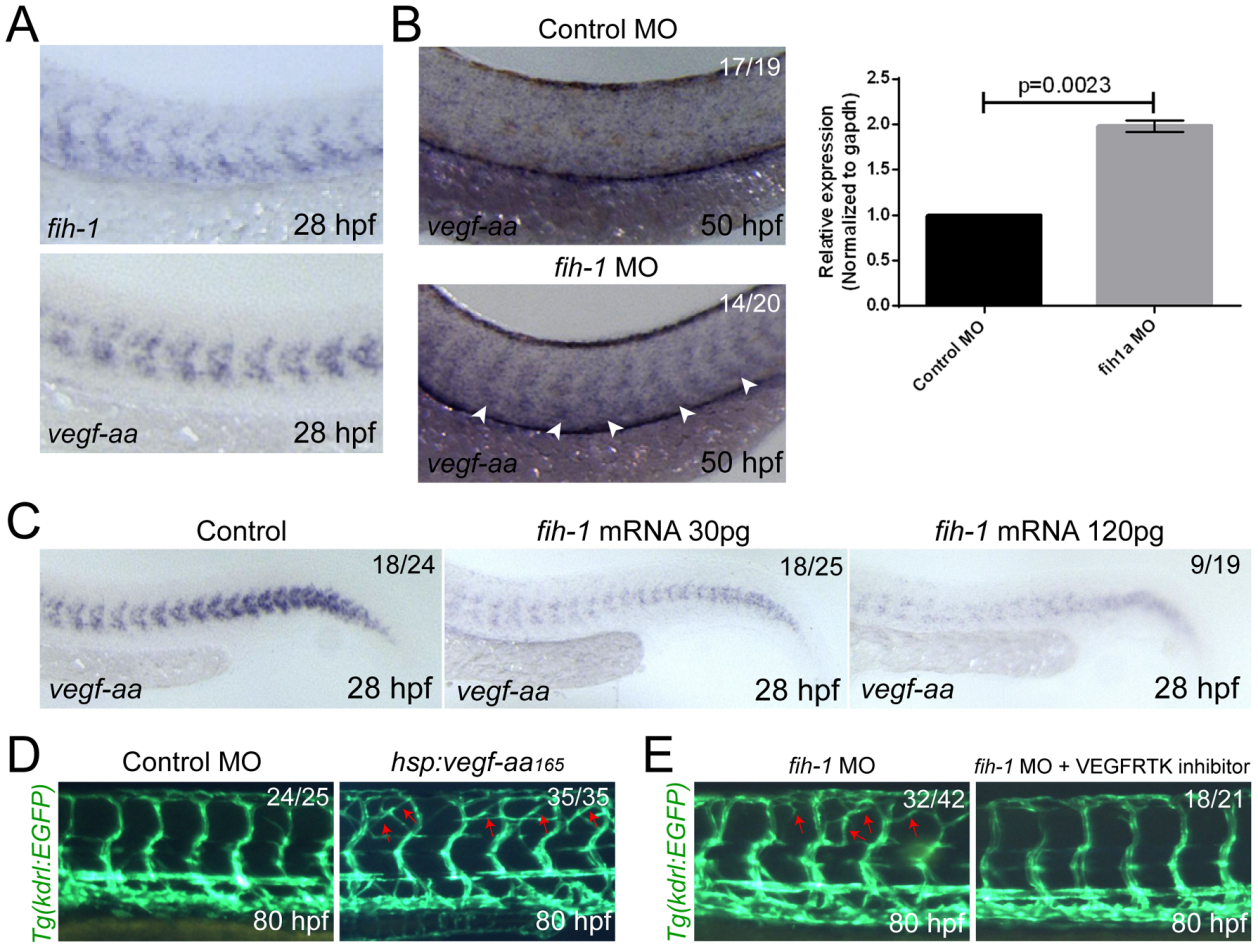
Fih-1 negatively regulates Vegf-aa_165_ in zebrafish embryos. (**A**) At 28 hpf, both *fih-1* and *vegf-aa_165_* transcript are selectively expressed within the anterior somites and ventral mesoderm. (**B**) Attenuation of Fih-1 activity in zebrafish elevates the level of *vegf-aa_165_* expression at 50 hpf. Arrowheads point to *vegf-aa_165_* expression in somites caused by lack of Fih-1 activity. Similarly, the level of *vegfaa* transcript in 48 hpf control or *fih-1* MO-injected embryos was evaluated by quantitative RT-PCR. Error bars are standard deviation (right). (**C**) Ectopic expression of Fih-1 decreases the level of *vegf-aa_165_* expression in a dose-dependent manner at 28 hpf. (**D**) Functional relationship between Fih-1 and Vegf-aa_165_. Ectopic expression of Vegf-aa_165_ under the regulation of *hsp70l* promoter caused a similar vascular phenotype as observed in *fih-1* MO-injected embryos. Moreover, inhibition of Vegf-A signaling can drastically reduce the exuberant angiogenic sprouts in *fih-1* MO-injected embryos at 80 hpf. Arrows point to ectopic sprouts.

Since FIH-1 negatively regulates HIF-1α, which in turn activates VEGF-A activity [20], [23], it is possible that the phenotype caused by the lack of FIH-1 activity may increase the transcription of *vegfa*. An elevated level of previously identified transcriptional targets of HIF-1α such as *hmox1a* [32] and *glut3* [33] in *fih-1* MO-injected embryos also supports this notion (Fig. S4). To examine whether the phenotype caused by the lack of FIH-1 activity (e.g. ectopic ISVs) may be induced by an elevated level of VEGF-A signaling, we examined the expression of *vegf-aa_165_*. Consistent with this idea, we find that *vegf-aa_165_*, a transcript encoding the predominant VEGF-A isoform, and *fih-1* express in a complementary manner in wild-type embryos at 28 hpf (Fig. 4A). In addition, the expression level of *vegf-aa_165_* is substantially elevated in *fih-1* MO-injected embryos (Fig. 4B), while drastically reduced in *fih-1* mRNA-injected embryos (Fig. 4C). Therefore, it appears that the expression level of *vegf-aa_165_* appears to be negatively regulated by FIH-1 during vascular development.

To further elucidate the functional relationship between VEGF-A165 and FIH-1, we generated a transgenic zebrafish line that expresses *vegf-aa_165_* under the regulation of a *hsp70l* promoter, *Tg(hspl:vegfaa165)^ck4^* (Fig. S5). The ectopic expression of *vegf-aa_165_* was induced at 50 hpf, and the resulting vascular phenotype was analyzed at 80 hpf. All *vegf-aa_165_* over-expressing embryos possess ectopic ISVs, similar to vascular defects in *fih-1* MO-injected embryos (Fig. 4D, red arrows). Conversely, treating *fih-1* MO-injected embryos with VEGF receptor tyrosine kinase inhibitor (VEGFTKI, Calbiochem), a small chemical antagonist of VEGF-A signaling, drastically reduces the ectopic ISVs (Fig. 4D). Taken together, our data suggest that FIH-1 may modulate vascular development by regulating VEGF-A165 activity via HIF-1α.

### Augmented FIH-1 activity suppresses angiogenesis in zebrafish embryos

**Figure 5:**
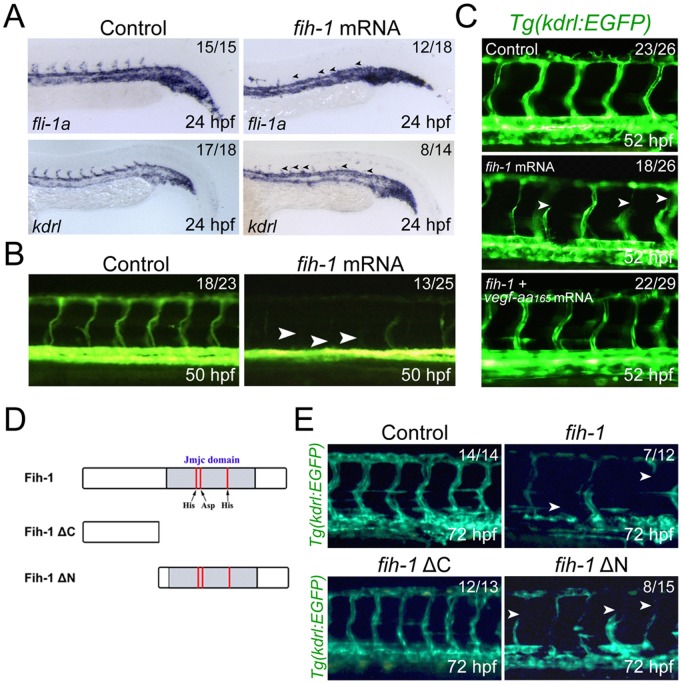
Over-expression of Fih-1 inhibits the formation of angiogenic vessels during development. (**A**) Whole-mount *in situ* hybridization with *fli1*a and *kdrl* in control or *fih-1* mRNA-injected embryos. In fih-1 mRNA-injected embryos, the number of intersegmental vessels (black arrowheads) was substantially reduced. (**B**) Microangiography of *fih-1* mRNA-injected embryos shows a lack of circulation in intersegmental vessels. (**C**) Ectopic expression of *vegf-aa_165_* restored impaired angiogenic vessel formation in *fih-1* mRNA-injected embryos at 52 hpf. Arrowheads point to intersegmental vessels which fail to anastomose at the dorsal-most part of the embryos. (**D**) Schematic diagram of two constructs that encode either N-terminus or C-terminus deleted Fih-1. Both N-terminus only Fih-1 (Fih-1ΔC) and C-terminus only Fih-1 (Fih-1ΔN) contain JmjC domain and metal binding sites which are essential for hydroxylation of HIF-1α. (**E**) C-terminus of Fih-1 is essential for its anti-angiogenic activity. While full length *fih-1* or *fih-1ΔN* mRNA-injected embryos inhibit angiogenic sprouts of intersegmental vessels, *fih-1ΔC* mRNA-injected embryos at 72 hpf do not show any obvious vascular defects. Arrowheads point to angiogenic sprouts which fail to extend to the dorsal-most side of the embryos.

Our data suggest that FIH-1 is a negative regulator of VEGF-A165 activity during development. In this scenario, the level of FIH-1 and VEGF-A165 activity will be inversely proportional. To examine whether an elevated level of FIH-1 can attenuate VEGF-A165 -mediated angiogenesis, we next expressed *fih-1* mRNA in wild-type embryos. In *fih-1* mRNA-injected embryos, the endothelial identity does not appear to be compromised (Fig. 5A). However, we find that a significant fraction of *fih-1* mRNA-injected embryos failed to develop intersegmental vessels (ISVs), whose formation is directly regulated by VEGF-A signaling (Fig. 5A). Microangiography also indicates a lack of circulation within ISVs in embryos injected with *fih-1* mRNA (Fig. 5B), further indicating the function of FIH-1 as a negative modulator for vascular development in zebrafish embryos. Co-injection of *vegf-aa_165_* mRNA was able to rescue angiogenic defects in the ISVs caused by *fih-1* mRNA-injection (Fig. 5C), substantiating the idea that balance between FIH-1 and VEGF-A165 activity is essential to modulate vascular development in zebrafish embryos. Taken together, our data suggest that FIH-1 negatively regulates VEGF-A signaling via HIF-1α.

Previously, it has been reported that FIH-1 can directly bind to VHL and HIF-1α via distinct domains, and form a ternary complex [20]. Therefore, identifying the essential domains for anti-angiogenic activity of FIH-1 *in vivo* may provide additional information on how FIH-1 and HIF-1α interact. We generated two truncated FIH-1 constructs, which lack either the C-terminus (FIH-1ΔC) or N-terminus (FIH-1ΔN) of FIH-1 (Fig. 5D). As shown previously in Fig. 5C, injection of full length FIH-1 severely disrupted the formation of ISVs (Fig. 5E). Injection of FIH-1ΔN construct led to similar vascular defects, while FIH-1ΔC failed to cause a similar phenotype (Fig. 5E). Therefore, it appears that the C-terminus is essential for the anti-angiogenic effects of FIH-1. Considering that the C-terminus of FIH-1 contains essential domains for its interaction with HIF-1α, our data indicate that anti-angiogenic effects of FIH-1 may be mediated by its interaction with HIF-1α during development.

## Discussion

In this report, we show that FIH-1 physically interacts with Mib, and functions as an anti-angiogenic factor by attenuating HIF-1α and VEGF-A signaling. Inhibition of FIH-1 in zebrafish embryos leads to ectopic angiogenesis from the ISVs, while over-expression of FIH-1 attenuates formation of ISVs, indicating that FIH-1 functions as an anti-angiogenic cue during development. Interestingly, global deletion of *Fih-1* in mouse does not cause any obvious vascular defects, but causes an elevated level of metabolism [34]. We believe different expression patterns of *fih-1* and *vhl* in zebrafish and mouse embryos can provide a potential explanation for this seemingly perplexing discrepancy. In zebrafish embryos, both *fih-1* and *vhl* expression can be strongly detected at the 4-cell stage, hinting that these transcripts are maternally deposited. Moreover, zygotic expression of *fih-1* precedes *vhl* and *fih-1* expression appears to be more abundant. Consistently, MO-mediated knock down of *hif1an* and *vhl* leads to similar vascular defects [35]. In contrast, mouse *fih-1* starts to express at E12.5 [34], two days later than the initiation of *vhl* expression [36]. We find that the onset of phenotypic defects in *fih-1* over-expressing embryos appear to be earlier than in *fih-1* MO-injected embryos. Since we utilized splicing MO to attenuate the translation of *fih-1* mRNA, it is possible that maternally deposited *fih-1* mRNA may undergo translation and ameliorate the knock-down phenotype. Alternatively, FIH-1 may have yet unidentified targets which may influence vascular development.

Previously, Notch signaling has been implicated as the main effector of Mib function during vascular development since Mib regulates endocytosis of Delta, a ligand for Notch [15]. As previously reported [12], we found that *mib* ^−/−^ embryos have ectopic vessels potentially due to excessive angiogenesis. Interestingly, *vegfaa* expression was unaltered in these embryos (Fig. S6), suggesting that inhibition of Notch signaling may modulate the angiogenic response independently to Vegf-Aa expression.

Considering the nature of FIH-1, it is tantalizing to postulate that Mib and FIH-1 mutually serve as a substrate for each other’s enzymatic activity, which creates a double negative feedback loop. By hydroxylating Mib, FIH-1 may promote degradation of Mib and inhibit Notch signaling, therefore promoting angiogenesis. In turn, active FIH-1 can facilitate degradation of HIF-1α, and attenuate the level of VEGF-A signaling, which negatively impacts angiogenesis. However, in *fih-1* MO-injected embryos, expression of *grl* and *dll4*, known targets of Notch signaling, appears to be unaltered (Figure S7), suggesting that FIH-1 may regulate HIF-1α to regulate the Vegf-Aa expression, but is unlikely to influence the expression of canonical Notch targets Further analyses on the interaction between FIH-1 and Mib would address the functional consequence of the their association in vascular development. Taken together, our data suggest that FIH-1 may function as an anti-angiogenic factor during vascular development by modulating VEGF-A signaling. Considering that the developmental function of anti-angiogenic factors are relatively unknown, our analyses on FIH-1 may provide novel insight on vascular development. Moreover, we find FIH-1 is physically associated with Mib, a known regulator of Notch signaling.

## Supporting Information

Figure S1 **Schematic diagram of yeast two-hybrid screen.** (**A**) Schematic diagram of the Mindbomb1 structure. Ankyrin repeats (AA410–744) from Mib protein was used as bait for yeast two hybrid screen. (**B**) List of potential interactors of Mib identified from the yeast two hybrid screen. (**C**) Strategy for yeast two hybrid screen.
(TIF)

Figure S2 **Specification of endothelial cells is not affected in** ***fih-1*** **MO-injected embryos.** Micrographs of whole-mount *in situ* hybridization with *fli-1a* (top rorw) and *scl* (bottom row) in control (left column) or *fih-1* (right column) MO-injected embryos at 12 hpf.
(TIF)

Figure S3 **Fih-1 regulates endothelial cell proliferation.** Proliferating endothelial cells in control (**A** and **C**) or *fih-1* MO-injected (**B** and **D**) at 55 hpf (**A** and **B**) and 72 hpf (**C** and **D**). The number of BrdU positive endothelial cells within the intersegmental vessels (ISVs) was significantly increased in *fih-1* MO-injected embryos at 55 and 72 hpf, compared to control embryos. Arrows indicate GFP^+^/BrdU^+^ endothelial cells in *Tg(kdrl:EGFP)* transgenic zebrafish (B'' and D''). Quantification on the number of GFP^+^/BrdU^+^ endothelial cells are shown in **E**. Asterisks indicate statistical significance (* p<0.005). Error bars, ±SD. n = 6 (55 hpf control), 5 (72 hpf control), 8 (55 hpf *fih-1* MO-injected), and 9 (72 hpf *fih-1* MO-injected).
(TIF)

Figure S4 ***fih-1*** **regulates** ***hif-1a*** **targets during zebrafish development.** (**A**) Schematic diagram on negative regulation of Hif-1α by Fih-1. Whole-mount *in situ* hybridization of *heme oygenase1a* (*hmox1a*) (**B**) and *glucose transporter-3* (*glut3*) (**C**) in control or *fih-1* MO-injected embryos. Lack of Fih-1 strongly induces expression of known Hif-1α targets.
(TIF)

Figure S5 **Generation of** ***Tg(hsp70l:vegfaa165)^ck4^*** **.** (**A**) Schematic diagram of the construct used to generate the *Tg(hsp70l:vegfaa165)* ***^ck4^*** zebrafish line. 12 hpf *Tg(hsp70l:vegfaa165)* ***^ck4^*** embryos without heat-shock (**B**) or with heat-shock (**C**). Strong induction of *vegfaa165* can be detected upon heat-shock treatment.
(TIF)

Figure S6 ***vegfaa*** **expression was not altered in** ***mib^−/−^*** **embryos.** (**A** and **B**) Expression of *vegfaa* was evaluated by whole mount *in situ* hybridization at 25 hpf in wild-type and *mib^−/−^* embryos.
(TIF)

Figure S7 **Endothelial notch target genes were not changed in** ***fih-1*** **MO-injected embryos. (A–D)** Expression of *gridlock (grl)* and *delta lik-4 (dll4)* were evaluated by whole mount *in situ* hybridization at 25 hpf in control and *fih-1* MO-injected embryos.
(TIF)
