# Supplementary figures and images for: FIH-1, a Novel Interactor of Mindbomb, Functions as an Essential Anti-Angiogenic Factor during Zebrafish Vascular Development
Source: PLoS One. 2014 Oct 27;9(10):e109517. doi: 10.1371/journal.pone.0109517 (PMC4209967; doi:10.1371/journal.pone.0109517)

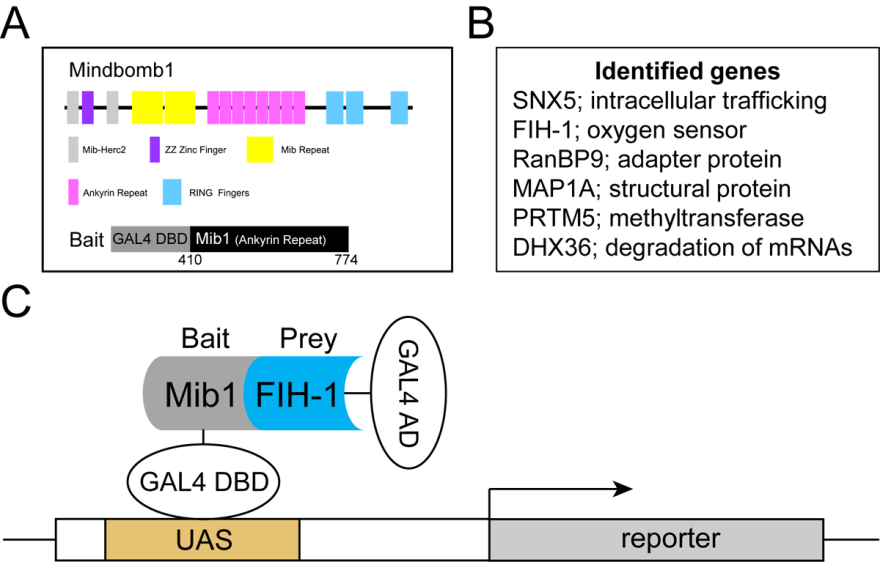

Supplement: Figure S1 — Schematic diagram of yeast two-hybrid screen. (A) Schematic diagram of the Mindbomb1 structure. Ankyrin repeats (AA410–744) from Mib protein was used as bait for yeast two hybrid screen. (B) List of potential interactors of Mib identified from the yeast two hybrid screen. (C) Strategy for yeast two hybrid screen. (TIF) [file pone.0109517.s001.tif]

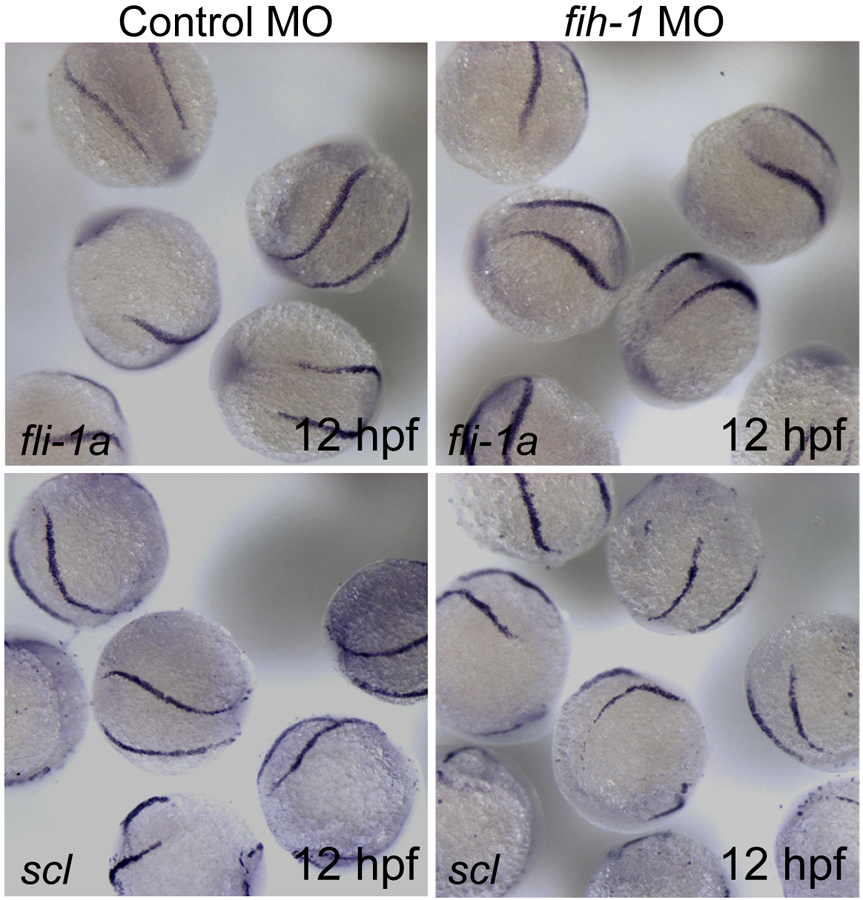

Supplement: Figure S2 — Specification of endothelial cells is not affected in fih-1 MO-injected embryos. Micrographs of whole-mount in situ hybridization with fli-1a (top rorw) and scl (bottom row) in control (left column) or fih-1 (right column) MO-injected embryos at 12 hpf. (TIF) [file pone.0109517.s002.tif]

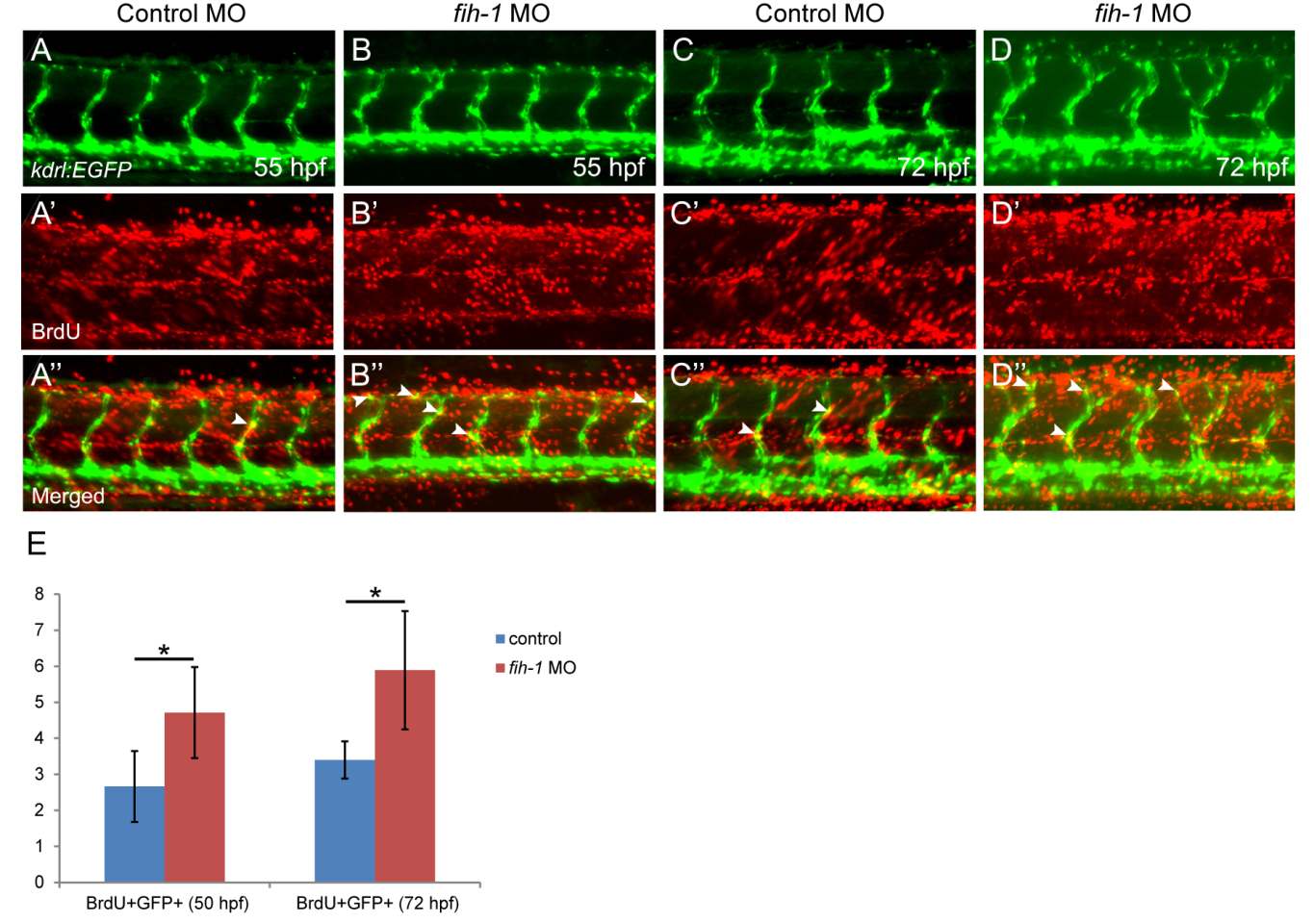

Supplement: Figure S3 — Fih-1 regulates endothelial cell proliferation. Proliferating endothelial cells in control (A and C) or fih-1 MO-injected (B and D) at 55 hpf (A and B) and 72 hpf (C and D). The number of BrdU positive endothelial cells within the intersegmental vessels (ISVs) was significantly increased in fih-1 MO-injected embryos at 55 and 72 hpf, compared to control embryos. Arrows indicate GFP+/BrdU+ endothelial cells in Tg(kdrl:EGFP) transgenic zebrafish (B'' and D''). Quantification on the number of GFP+/BrdU+ endothelial cells are shown in E. Asterisks indicate statistical significance (* p<0.005). Error bars, ±SD. n = 6 (55 hpf control), 5 (72 hpf control), 8 (55 hpf fih-1 MO-injected), and 9 (72 hpf fih-1 MO-injected). (TIF) [file pone.0109517.s003.tif]

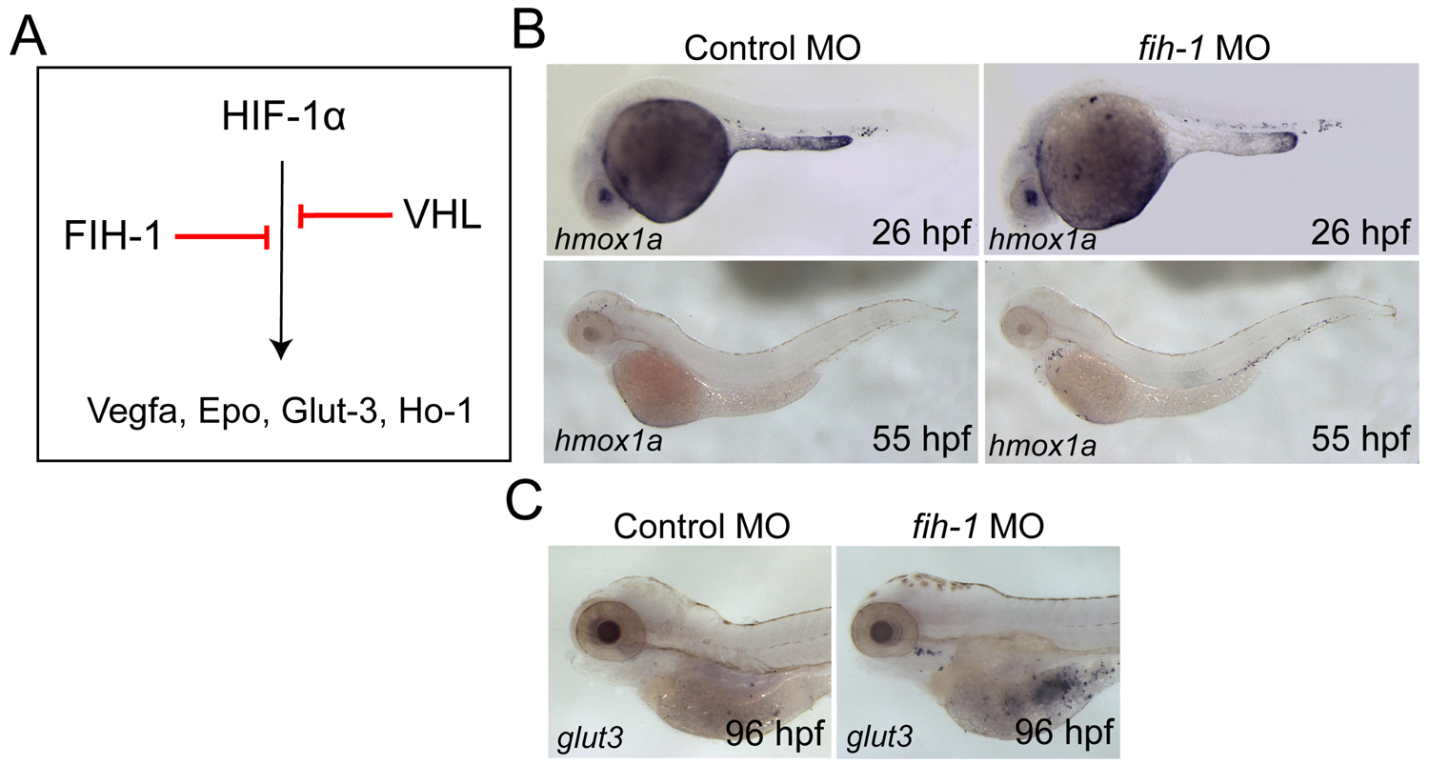

Supplement: Figure S4 — fih-1 regulates hif-1a targets during zebrafish development. (A) Schematic diagram on negative regulation of Hif-1α by Fih-1. Whole-mount in situ hybridization of heme oygenase1a (hmox1a) (B) and glucose transporter-3 (glut3) (C) in control or fih-1 MO-injected embryos. Lack of Fih-1 strongly induces expression of known Hif-1α targets. (TIF) [file pone.0109517.s004.tif]

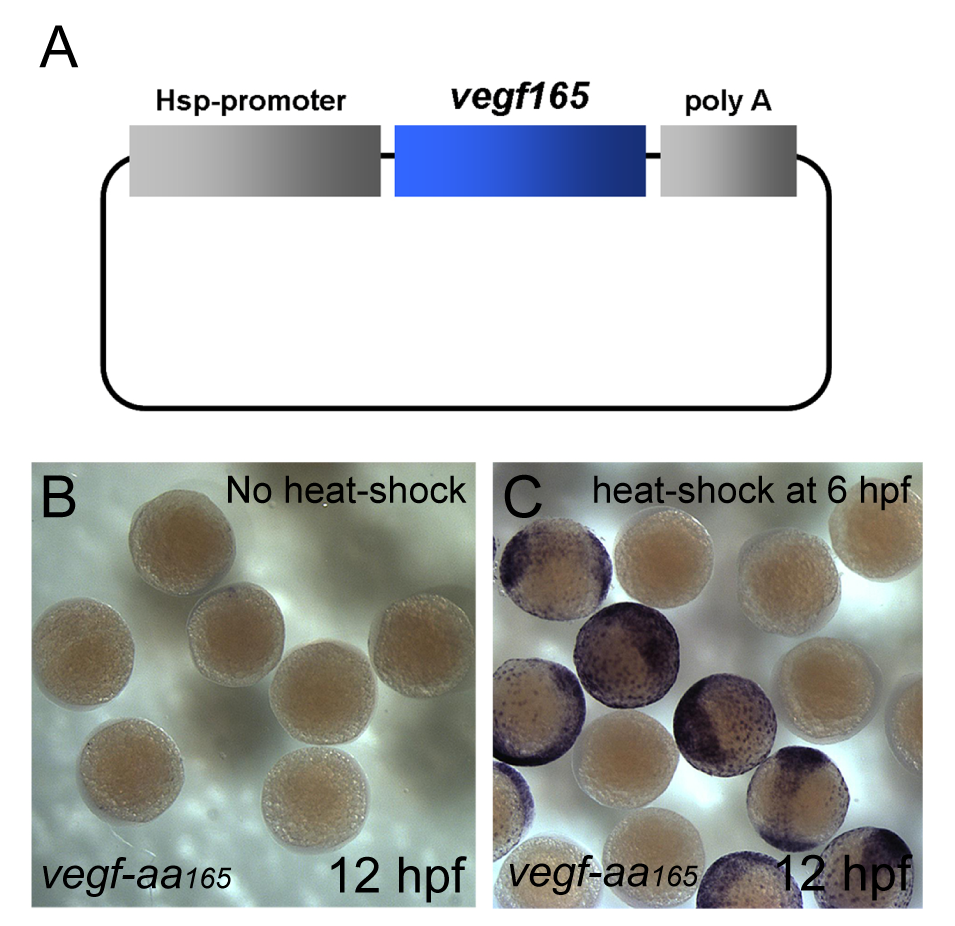

Supplement: Figure S5 — Generation of Tg(hsp70l:vegfaa165)ck4 . (A) Schematic diagram of the construct used to generate the Tg(hsp70l:vegfaa165) ck4 zebrafish line. 12 hpf Tg(hsp70l:vegfaa165) ck4 embryos without heat-shock (B) or with heat-shock (C). Strong induction of vegfaa165 can be detected upon heat-shock treatment. (TIF) [file pone.0109517.s005.tif]

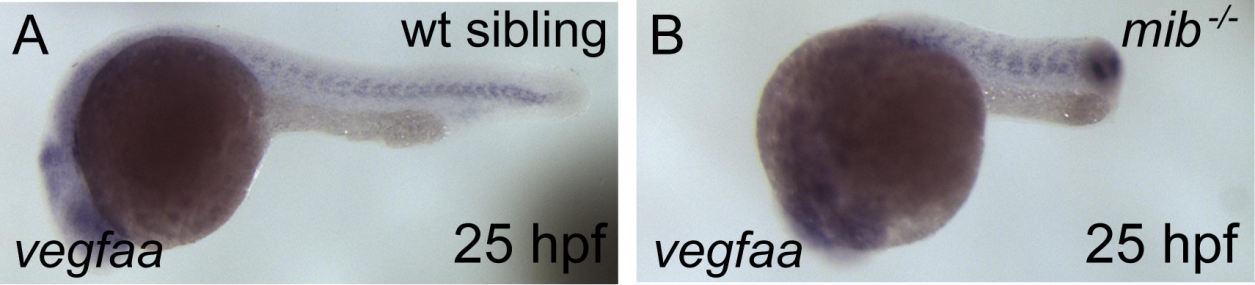

Supplement: Figure S6 — vegfaa expression was not altered in mib−/− embryos. (A and B) Expression of vegfaa was evaluated by whole mount in situ hybridization at 25 hpf in wild-type and mib−/− embryos. (TIF) [file pone.0109517.s006.tif]

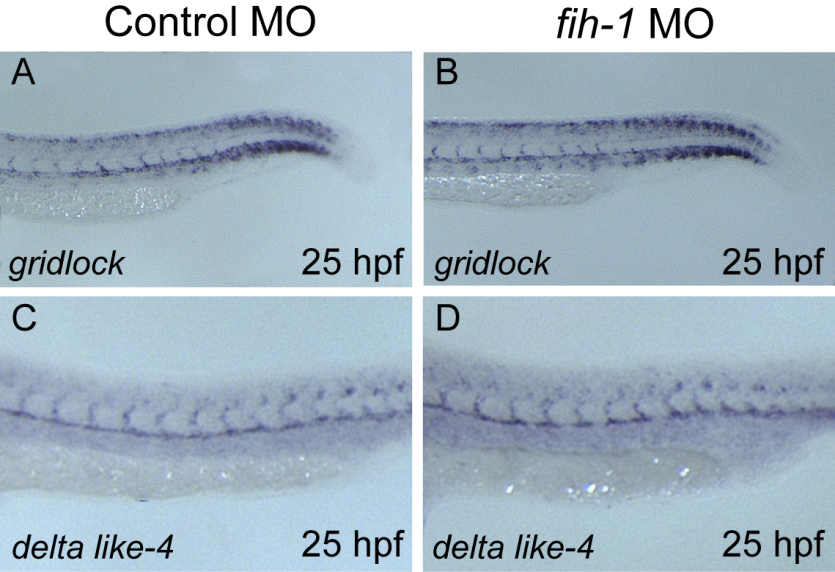

Supplement: Figure S7 — Endothelial notch target genes were not changed in fih-1 MO-injected embryos. (A–D) Expression of gridlock (grl) and delta lik-4 (dll4) were evaluated by whole mount in situ hybridization at 25 hpf in control and fih-1 MO-injected embryos. (TIF) [file pone.0109517.s007.tif]
